# Letter on: “Assessment of heart rate variability and occurrence of falls in Alzheimer's disease: an exploratory study”

**DOI:** 10.1055/s-0046-1827045

**Published:** 2026-09-25

**Authors:** Etika Rana, Sanam Maurya

**Affiliations:** 1 Maharishi Markandeshwar Institute of Physiotherapy and Rehabilitation, Maharishi Markandeshwar (Deemed to be University) Mullana, Ambala HR, India.

Dear Editor,

We read with interest the study by Rodrigues et al. 1 exploring the relationship between heart rate variability (HRV) and falls in individuals with Alzheimer's disease (AD). The topic is clinically important; however, several methodological and analytical aspects warrant careful consideration when interpreting the findings.

Firstly, the study is based on a small final sample (n = 26), following multiple exclusions related to signal quality and participant-related factors. 1 Such exclusions may introduce selection bias, particularly if excluded individuals differ in clinically meaningful ways from those included. Additionally, small sample sizes reduce statistical power and may lead to unstable or imprecise estimates. 2 The absence of a priori sample size calculation further limits confidence in whether the study was adequately powered.

Secondly, the cross-sectional design, combined with retrospective assessment of falls over a 3-year period, restricts causal interpretation. Falls are dynamic events influenced by changes in health status over time, and long-term recall is known to be unreliable. 3 As a result, the temporal relationship between HRV alterations and fall events remains uncertain.

Thirdly, potential confounding factors have not been sufficiently addressed. Differences in sedentary behavior and medication use—particularly neuroleptics—were evident between groups. These factors are independently associated with both autonomic dysfunction and fall risk. However, the analysis relies on unadjusted comparisons without multivariable modelling, making it difficult to determine whether the observed associations are independent or confounded. 4

Fourthly, the statistical approach raises concerns regarding multiplicity and consistency. A large number of HRV parameters were analyzed across multiple domains and conditions (Tables 2–5 from their study), 1 without adjustment for multiple comparisons. This increases the risk of chance findings, particularly in small samples. 5 Additionally, both parametric and non-parametric tests were applied without clear justification of underlying assumptions, which may affect the validity of the results.

Finally, the reporting of results is limited by reliance on *p* -values, without accompanying effect sizes or confidence intervals. Such measures are important for understanding the magnitude and precision of associations. 4 Furthermore, falls were assessed retrospectively as a binary outcome rather than using prospective or standardized methods, which may reduce measurement accuracy. 3

In summary, while the study provides useful preliminary observations, the findings may be considered exploratory, and attention to the methodological and analytical aspects outlined above may help to better interpret the relationship between HRV and falls in AD.
